# Effectiveness of a Mobile Short-Message-Service–Based Disease Outbreak Alert System in Kenya

**DOI:** 10.3201/eid2204.151459

**Published:** 2016-04

**Authors:** Mitsuru Toda, Ian Njeru, Dejan Zurovac, Shikanga O Tipo, David Kareko, Matilu Mwau, Kouichi Morita

**Affiliations:** Japan International Cooperation Agency, Tokyo, Japan (M. Toda);; Nagasaki University Institute of Tropical Medicine, Nagasaki, Japan (M. Toda, K. Morita);; Ministry of Health Kenya, Nairobi, Kenya (I. Njeru, S. O-Tipo, D. Kareko);; Kenya Medical Research Institute Wellcome Trust Research Programme, Nairobi (D. Zurovac);; Oxford University, Oxford, UK (D. Zurovac);; Kenya Medical Research Institute, Nairobi (M. Mwau)

**Keywords:** cell phones, developing countries, communicable diseases, randomized controlled trial, disease notification, disease outbreaks, public health surveillance, epidemiology, Kenya, bioterrorism and preparedness

## Abstract

We conducted a randomized, controlled trial to test the effectiveness of a text-messaging system used for notification of disease outbreaks in Kenya. Health facilities that used the system had more timely notifications than those that did not (19.2% vs. 2.6%), indicating that technology can enhance disease surveillance in resource-limited settings.

Outbreaks of epidemic diseases pose serious public health risks (*1*). Kenya, like other Africa countries, lacks the means to deliver adequate healthcare services. This weakness compromises the success of the World Health Organization’s Integrated Disease Surveillance and Response (IDSR) and International Health Regulations (IHR) strategies and often results in incomplete, delayed, and poor-quality (i.e., not following standard case definitions in the IDSR guidelines) paper-based reporting from health facilities in remote areas. Furthermore, inadequate reporting limits health managers’ ability to take appropriate and timely action in response to health events (*2**,**3*). 

Widespread expansion of mobile phone coverage in Africa (*4*) offers opportunities to overcome weaknesses in health systems and to improve medical and public health practice through mobile health (mHealth) (*5*). Despite many mHealth projects undertaken in Africa, their effectiveness has rarely been rigorously evaluated, limiting evidence-based policy adoptions or project expansion in scope or geography (*6**–**9*). In particular, evidence of effectiveness of mHealth interventions for enhancing disease surveillance is scarce (*10*). We undertook a clustered, randomized, controlled trial with 135 health facilities in Busia and Kajiado Counties in Kenya during November 2013–April 2014 to test the effectiveness of a mobile short-message-service (SMS)–based disease outbreak alert system (mSOS) for reporting immediately notifiable diseases.

## The Study

**Figure 1:**
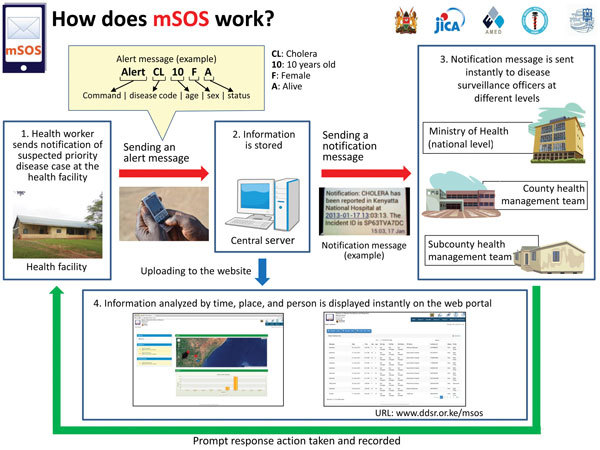
Structure and communication flow of a mobile short-message-service–based disease outbreak alert system (mSOS) in Kenya. Source: mSOS Technical Working Group, Ministry of Health Kenya.

mSOS is a formatted text-messaging system that enables communications between healthcare facility workers and Ministry of Health managers and uses a Web-based portal to monitor disease notifications and response actions taken by health managers (Figure 1; online Technical Appendix, http://wwwnc.cdc.gov/EID/article/22/4/15-1459-Techapp1.pdf). In our trial, health workers used mSOS for 6 months to send information about suspected cases or health events that required notification within 24 hours. Twelve diseases and conditions were selected for the study (online Technical Appendix Table 1). Before mSOS was implemented, we conducted a 1-day refresher training course on IDSR for in-charges (i.e., medical officers in charge) of 135 participating health facilities; the training focused on case definitions of notifiable diseases and on paper-based reporting. During the training, facilities were randomized into intervention and control groups; the intervention group received an additional day of training on mSOS. Paper-based reporting continued throughout the study period for both groups, so the intervention group would report cases 2 ways.

**Table 1:** Characteristics of health facilities and their in-charges for intervention and control groups and study periods, Kajiado County, Kenya*

Characteristic	Preintervention, no. (%)			Postintervention, no. (%)		
	Control, N = 65	Intervention, n = 66		Control, n = 65	Intervention, n = 66	p value†
Health facilities, Kajiado County	42 (64.6)	41 (62.1)		42 (64.6)	41 (62.1)	0.767
Ownership						
Public	39 (60.0)	45 (68.2)		39 (60.0)	45 (68.2)	0.329
Private	15 (23.1)	13 (19.7)		15 (23.1)	13 (19.7)	0.637
FBO/NGO	11 (16.9)	8 (12.1)		11 (16.9)	8 (12.1)	0.435
Level of care						
Hospital/health center	20 (30.8)	19 (28.8)		20 (30.8)	19 (28.8)	0.804
Dispensary	40 (61.54)	43 (65.15)		40 (61.5)	43 (65.2)	0.668
Other facility	5 (7.7)	4 (6.1)		5 (7.7)	4 (6.1)	0.712
Resource availability						
Mobile phone	65 (100)	66 (100)		65 (100)	66 (100)	–
Electricity	45 (69.2)	47 (71.2)		54 (83.1)	49 (74.2)	0.217
Water	54 (83.1)	47 (71.2)		51 (78.5)	50 (75.8)	0.713
Surveillance focal person	48 (73.9)	44 (67.7)		44 (67.7)	47 (71.2)	0.662
IDSR reporting tool‡	22 (33.9)	23 (34.9)		34 (52.3)	32 (48.5)	0.662
IDSR job aid	44 (67.7)	44 (66.7)		49 (75.4)	55 (83.3)	0.261
Characteristic of in-charge						
Female sex	32 (49.2)	39 (59.1)		32 (49.2)	39 (59.1)	0.257
Median age, y (IQR)§	34 (29–48)	35 (30–42)		36 (30–49.5)	37 (30–44)	0.677
Doctor/clinical officer	12 (18.5)	15 (22.7)		16 (24.6)	13 (19.7)	0.498
Nurse	46 (70.8)	48 (72.7)		44 (67.7)	48 (72.7)	0.529
Other healthcare worker	7 (10.8)	3 (4.6)		5 (7.7)	5 (7.6)	0.980

*The table does not show data for Busia County because values will be inverse of data for Kajiado County (i.e., N minus n). N = total facilities in both counties. The intervention group is the group of facility in-charges who were exposed to IDSR and mSOS training and to the mSOS intervention; the control group is the group of in-charges who were exposed to IDSR training only. FBO, faith-based organization; IDSR, Integrated Disease Surveillance and Response; in-charge, medical officer in charge of facility; IQR, interquartile range; NGO, nongovernment organization. †χ^2 ^test was used to compare the proportions between control and intervention groups. Wilcoxon Mann Whitney test was used to compare medians between control and intervention groups (i.e., age of in-charges). Analyses were conducted by using an α level of 0.05. The p value is shown for the postintervention period only. ‡Standardized IDSR paper-based reporting form for immediately notifiable diseases. §Data are median and Interquartile range rather than numbers and percentages. Denominator excludes 3 facilities with missing values in the preintervention control group and 1 facility with missing values for each of the remaining 3 study groups.

**Figure 2:**
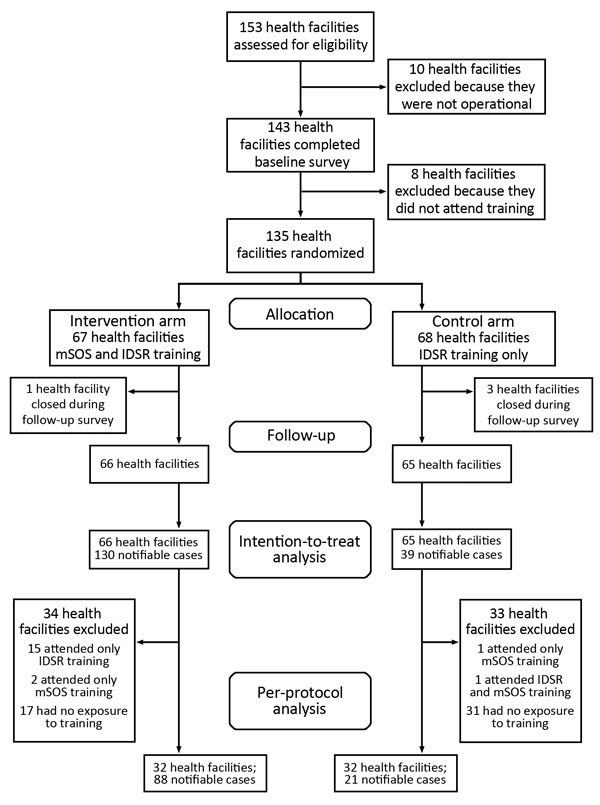
Profile of control and intervention health facilities and exclusions during the course of a study of a mobile short-message-service–based disease outbreak alert system (mSOS) in Kenya. IDSR, Integrated Disease Surveillance and Response.

Our primary outcome was determining how many of the cases that required immediate notification were reported within the time specified. Our secondary outcome was determining, from among the cases for which notifications were sent, the proportion for which response actions were taken. For evaluation purposes, data from health facilities were collected for 6-month periods before and after the intervention launch (i.e., IDSR and mSOS training and use of mSOS for 6 months). Cases detected, notifications submitted, and responses undertaken were extracted from facility records in both study groups. Notifications sent by SMS were retrieved from the mSOS system. Our primary analysis was intention-to-treat (i.e., analysis of cases from all health facilities as they were randomized, regardless of intervention exposure). Our secondary analysis was per-protocol (i.e., our trial protocol) and was restricted to cases reported by facilities whose in-charges had received training (i.e., IDSR training for control group; IDSR and mSOS training for intervention group; Figure 2).

Characteristics of health facilities and in-charges were similar; data from preintervention and postintervention surveys showed no significant differences between control and intervention groups (Table 1). Follow-up surveys conducted 6 months after the intervention showed that 34 (51.6%) of 66 intervention group in-charges received mSOS and IDSR training and 32 (49.2%) of 65 control group in-charges received IDSR training (Figure 2; online Technical Appendix). 

A retrospective review of the baseline (preintervention) surveys showed that 36 cases (19 for intervention group, 17 for control group), all measles, required immediate notification. Of these 36 cases, only 1 immediately notifiable case was reported (from a control facility using paper forms). During the 6-month period after the intervention, 169 immediately notifiable cases (130 for the intervention group, 39 for the control group) were detected: 160 measles, 6 anthrax, 2 Q fever, and 1 guinea worm. Of the 39 cases detected in the control group, notification of only 1 case (2.6%), which was measles, was sent. Of the 130 immediately notifiable cases detected in the intervention group, 25 (19.2%) were reported to disease surveillance coordinators at the subcounty, county, and national levels. This proportion of cases reported was significantly higher than that reported by the control group (% difference 16.7, 95% CI 2.71–25.07; Table 2). 

**Table 2:** Postintervention reporting of immediately notifiable cases by study group under the intention-to-treat and per-protocol analysis*

Type of analysis	Control			Intervention		% Difference (95% CI)
	Total	Cases notified, no. (%)		Total	Cases notified, no. (%)	
Intention to treat	39	1 (2.6)		130	25 (19.2)	+16.7 (2.71–25.07)
Per protocol	21	1 (4.8)		88	24 (27.3)	+22.5 (−0.32 to 34.13)

*Intention-to-treat analysis indicates analysis of treatment groups as they were randomized, regardless of the intervention exposure; per-protocol analysis indicates restricted analysis of groups that completed the entire study according to the trial protocol.

All 25 cases for which notifications were sent from the intervention group were measles cases reported through mSOS; 2 cases were also reported with paper forms. For these 25 mSOS notifications, the threshold for a measles outbreak response (5 suspected cases) was met once, and disease surveillance coordinators at the subcounty level responded to this event. Furthermore, 24 (96%) of the 25 suspected measles cases were reported within 24 hours. 

In the per-protocol analysis, the percentage of cases for which notification was sent was greater in the intervention group than in the control group (27.3% vs. 4.8%), but the difference was of borderline statistical significance (% difference 22.5, 95% CI −0.32 to 34.13 by Wilson procedure with continuity correction [*11*]). Similar differences were found when the analysis was restricted to health facilities that stocked paper-based tools (i.e., control group, 1/18 [5.6%] vs. intervention group, 22/78 [22.6%]; % difference 17.0, 95% CI −2.93 to 35.30).

## Conclusions

This study showed that SMS intervention significantly increased timely notifications; however, despite a relatively large improvement, response remained suboptimal, with timely notifications of only one fifth of detected cases. These findings mirror results of a study in Tanzania, which showed that SMS considerably increased vital registration coverage but fell far short of reporting actual birth and death events in the community (*12*).

Our study has implications for health managers who implement interventions to improve disease surveillance in resource-limited settings. First, the number of detected cases requiring immediate notification increased postintervention. This effect was observed in both intervention and control groups but was higher in the group using SMS; this group had a 7-fold increase in detected cases compared with baseline findings. IDSR refresher training may have contributed to increased case detection, and the combined interventions, including the technology component, resulted in a greater detection effect. Second, expecting health workers to complete paper-based forms and deliver them without incentive within 24 hours is ineffective for ensuring notification of cases, with or without exposure to the refresher training. Third, we observed a large drop-out rate (47.4%) for health facility in-charges participating in the study. The study took place during a period of health management decentralization in Kenya, resulting in 47 new counties and in health worker transfers. Lack of on-the-job training for staff who did not attend the training and lack of support through posttraining follow-up and supportive supervision were weaknesses in the intervention. These systemic challenges, reported in other IDSR (*13*) and mHealth surveillance (*14*) projects, must be addressed to avoid compromising the sustainability of such interventions. Finally, attrition of health workers exposed to the intervention and lack of paper-based tools explain only part of our results. The short duration of the training deployed (*15*) and the possibly suboptimal quality of the training delivered (*3*) may have contributed to the unrealized full potential of the intervention.

Despite its limitations (online Technical Appendix), this study shows how technology in the form of mSOS can increase the rate of notifications of suspected disease outbreaks and enhance IHR compliance in resource-limited settings. Further investigation into ways to optimize the quality of delivery of mSOS interventions in countries with weak healthcare systems is justified.

**Technical Appendix.** Methods and additional details of a study of a mobile short-message-service–based disease outbreak alert system in Kenya.
