# Correction: Vol. 22, No. 4

**DOI:** 10.3201/eid2208.C12208

**Published:** 2016-08

**Keywords:** errata, erratum, correction

An affiliation for Mitsuru Toda was missing from the article Effectiveness of a Mobile Short-Message-Service–Based Disease Outbreak Alert System in Kenya (M. Toda et al.). The article has been corrected online (http://wwwnc.cdc.gov/eid/article/22/4/15-1459_article).

