# Supplementary material for: Effectiveness of a Mobile Short-Message-Service–Based Disease Outbreak Alert System in Kenya
Source: Emerg Infect Dis. 2016 Apr;22(4):711–5. doi: 10.3201/eid2204.151459 (PMC4806970; doi:10.3201/eid2204.151459)
Supplement: Supplementary file 1 — Technical Appendix. Methods and additional details of a study of a mobile short-message-service–based disease outbreak alert system in Kenya. [file 15-1459-Techapp-s1.pdf]

Article DOI: http://dx.doi.org/10.3201/eid2304.151459

Effectiveness of a Mobile Short-Message Service-Based Disease Outbreak Alert System in Kenya

Technical Appendix

Additional Details of the mSOS Study in Kenya

Detailed Methodology

Study Sites

The study took place at health facilities in Busia and Kajiado Counties in Kenya. These counties were selected because of historic records of outbreaks of viral hemorrhagic fevers (*1*,*2*). Busia County borders Uganda by the Victoria Lake basin; has a population of 740,043; covers a surface area of 1,134 km$^{2;}$ and is divided into 7 subcounties that represent first-level health management units in Kenya. Kajiado County, which borders Tanzania, has a population of 682,123; a surface area of 2,190 km^2; and 5 subcounties. The World Health Organization’s Integrated Disease Surveillance and Response (IDSR) was implemented in both counties in 2005. In alignment with national guidelines for reporting suspected immediately notifiable diseases, IDSR involves completing and submitting paper-based forms from rural health facilities in Kenya to the subcounty-level disease surveillance coordinators, who electronically transmit information to higher-level managers and provide the first-level response action to the reporting facilities (*3*).

Study Participants

Participants in the study included in-charges of health facilities that were registered on the official Ministry of Health Kenya Master Facility List (*4*) operational during the study period. These facilities provided curative services and were operated by government, faith-based, nongovernmental, or private organizations. Absence of a mobile phone network at the facility and the inability of facility in-charges to use short-message services (SMS) were exclusion criteria.

Intervention

The intervention was a mobile SMS-based disease outbreak alert system (mSOS), which was developed by the Ministry of Health (MOH) in collaboration with the Faculty of Information and Technology at Strathmore University in Nairobi and was pretested and refined at several health facilities before training and deployment began in the study areas. The mSOS consisted of formatted SMS communication between health workers at local facilities and MOH managers at the subcounty, county, and national levels. A web-based mSOS portal was developed and used to monitor notifications sent by health facility workers and response actions taken by the national disease surveillance officers and managers (Figure 1 in main text). Health workers used an mSOS text messaging system for 6 months to send patient-level information for suspected cases that required immediate (i.e., within 24 hours) notification. Twelve diseases and conditions listed in the national IDSR guidelines were selected for the study (Technical Appendix Table). Text messages sent by health workers consisted of prescribed codes specifying patients' disease diagnosis, age, sex, and survival status (i.e., alive or dead). The messages were sent to a toll-free number set up by a telecommunication provider in Kenya. Health managers at all levels received text messages in real time on their mobile phones. By using a password-protected web-based portal, they could also observe all notifications, maps with locations of health facilities where incidents occurred, and graphs showing cumulative cases reported. All information sent by mSOS was stored on a secure server at the MOH.

Before mSOS was implemented in the study areas, a 1-day IDSR refresher training for all in-charges of health facilities was conducted during September and October 2013. The training focused on case definitions of notifiable diseases and routine paper-based case notifications. During the training, health facilities were randomized into control or intervention groups, and participants from the intervention group facilities received an additional day of training on using mSOS. During the mSOS training for health workers, the subcounty and county disease surveillance coordinators were also trained on how to access and use the web-based portal to view mSOS information and how to log the response actions taken. Throughout the study period, the paper-based reporting, as indicated in the national IDSR guidelines, continued in both the intervention and control facilities; the intervention group was also trained to use mSOS to report the same cases reported by paper.

Randomization and Masking

Randomization was conducted during the IDSR training by stratifying health facilities by subcounties and randomly selecting intervention facilities from each stratum by using a 1:1 ratio. The intervention group was unmasked because of the nature of the study; investigators, health workers, and health mangers were observing SMS notifications and were aware of which study facilities were in the intervention group.

Data Collection

To evaluate the intervention, pre- and post-intervention surveys were undertaken by the health facilities. In June 2013, baseline retrospective data were collected for the 6-month period before the intervention (December 2012–May 2013). In May 2014, the data were collected for the six-month duration after the intervention was launched (November 2013–April 2014). At each study facility, trained data collectors reviewed all outpatient, inpatient, and maternal and child health registers and extracted patient-level information for the 12 diseases and conditions selected for the study. For each extracted case, date of patient’s visit, name, sex, age, and provisional diagnosis were recorded. Copies of submitted paper-based reports for immediately notifiable diseases were also reviewed. In addition, the visitors’ and supervision books signed by surveillance coordinators were reviewed at the health facility to determine whether any response action was taken at the health facility after the notification was sent.

For the intervention group, notifications sent through mSOS were also extracted. During the surveys, all in-charges of health facilities were interviewed, and information on characteristics of the facility and of the in-charges managing the facility and their exposure to the intervention was recorded. Data extracted from facility records were collected on paper forms, and data from structured interviews with facility in-charges were collected by using Magpi software (*5*) installed on data collectors’ mobile phones.

Definitions

A case requiring immediate notification was defined as any of 12 notifiable diseases and conditions extracted from any of the facility registers. Data from the source documents were examined to eliminate duplicate cases by using patient’s diagnosis, date, and name. Notifications were defined as cases reported through paper-based forms in the control group and mSOS or paper forms in the intervention group. A response action taken was defined as visits to the reporting facility by the subcounty, county, or national surveillance coordinators,

as documented in visitors' or supervision books in the control group or through the mSOS web portal or visitors' or supervision books in the intervention group. According to the national IDSR guidelines in Kenya, the 12 notifiable diseases and conditions, except for measles, required immediate notification within 24 hours of detection, and response action was required within 24 hours of notification. Measles required immediate notification within 24 hours of detection, and response action was required within 24 hours of notification of the fifth suspected measles case detected in the same health facility or subcounty during 1 month. The number of notification days was calculated as the period of days between the date of case detection and the date of notification.

Statistical Analysis

All analyses were performed by using Stata version 12 (College Station, Texas, USA; http://www.stata.com/). The primary analysis was "intention-to-treat" and included cases from all study facilities as the facilities were randomized, regardless of the intervention exposure. The secondary analysis was "per-protocol" (i.e., study protocol) and was restricted to the cases from the facilities where the facility in-charges were exposed to the IDSR training in the control group and to IDSR and mSOS training in the intervention group. An additional analysis in which per-protocol conditions were further restricted to the case-patients seen at health facilities with available paper-based tools was also performed. To explore potential confounders, the χ^2 test for proportions and the Wilcoxon Mann Whitney test for median comparisons were conducted on characteristics of health facilities and of their in-charges to compare the control and intervention groups. Because no significant differences were found and only 1 notified case across study groups was reported at baseline, results from the post-intervention survey under the intention-to-treat and per-protocol analyses were the primary focus of results (presented in this article. Because of the small population sizes for both analyses, which precluded cluster adjustments, we calculated the 95% CIs around differences in proportions between notification outcomes for the intervention and control groups by using the Wilson procedure with continuity correction (*6*,*7*). CI estimations were done at an α level of 0.05.

Ethical Considerations

Ethical approval was obtained from Kenya Medical Research Institute (KEMRI) Ethical Review Committee (SSC 2523). The trial is registered with Current Controlled Trials

ISRCTN 79529838. Written informed consent was obtained from all health facility in-charges enrolled in the study for baseline and follow-up surveys.

Trial Profile

Figure 2 shows the trial profile, including characteristics of nonexposure and contamination (i.e., when facility in-charges crossed from the intervention group to the control group or vice versa during the study period) of the trial facilities 6–8 months after delivery of the intervention. Before the study began, 153 health facilities from the Master Facility List were assessed for eligibility in the study areas. Ten facilities were excluded because they were nonoperational at the time of the study. The baseline survey was therefore undertaken at 143 health facilities. Of 143 facilities, in-charges of 135 facilities attended the training, where 67 and 68 facilities, respectively, were randomized into intervention and control groups. Four facilities had closed by the time the follow-up survey was undertaken 6–8 months later. The follow-up survey included 131 health facilities, of which 66 from the intervention group and 65 from the control group were included in the primary, intention-to-treat analysis. Of the 66 facilities in the intervention group, the follow-up survey showed 34 (51.6%) facilities with in-charges who received the complete intervention: both IDSR and mSOS training. The other in-charges in the intervention group either did not attend the study training (17 [25.7%]) or attended only either the IDSR component (15 [22.7%]) or the SMS component of the training (2 [3.0%]). As with the intervention group, only 32 (49.2%) of 65 facilities in the control group had in-charges who received routine IDSR training during the intervention delivery. Because of transfers of health workers, 2 facilities in the control group were also found to be contaminated with in-charges who were exposed to the SMS component of the intervention. The restricted per-protocol analysis included 64 health facilities (32 in the intervention group and 32 in the control group).

Limitations

This study has several possible limitations. First, we did not capture possible informal notifications through phone calls or in-person interactions between health facility in-charges and disease surveillance coordinators at the subcounty, county, and national levels. This lack of information may have underestimated the true rates of managers' awareness about immediately notifiable cases, but the information collected in the study reflects the true degree of compliance with the IDSR national guidelines. Second, the lack of completeness of notifiable cases recorded by health workers in the source documents at the health facilities is an inherent problem and may have resulted in selection bias. Such bias was partly remedied

through the randomized design. Finally, the study did not verify whether the diseases recorded in the source documents were correctly diagnosed or recorded on the basis of case definitions or laboratory confirmations; these verification measures were beyond the scope of the study.

References

1. Murithi RM, Munyua P, Ithondeka PM, Macharia JM, Hightower A, Luman ET, et al. Rift Valley fever in Kenya: history of epizootics and identification of vulnerable districts. Epidemiol Infect. 2011;139:372–80. http://dx.doi.org/10.1017/S0950268810001020

2. Kwallah A, Inoue S, Thairu-Muigai AW, Kuttoh N, Morita K, Mwau M. Seroprevalence of yellow Fever virus in selected health facilities in western Kenya from 2010 to 2012. Jpn J Infect Dis. 2015;68:230–4. http://dx.doi.org/10.7883/yoken.JJID.2014.288

3. Ministry of Public Health and Sanitation. Integrated Disease Surveillance and Response in Kenya: technical guidelines 2012; Nairobi (Kenya): The Ministry; 2012.

4. Ministry of Health, Kenya. Master health facility list: eHealth Kenya. 2011 [cited 2012 Oct 31]. http://www.ehealth.or.ke/facilities/

5. Magpi. 2015 [cited 2015 May 27]. http://home.magpi.com

6. Wilson EB. Probable inference, the law of succession, and statistical inference. J Am Stat Assoc. 1927;22:209–12. http://dx.doi.org/10.1080/01621459.1927.10502953

7. Newcombe RG. Interval estimation for the difference between independent proportions: comparison of eleven methods. Stat Med. 1998;17:873–90. http://dx.doi.org/10.1002/(SICI)1097-0258(19980430)17:8<873::AID-SIM779>3.0.CO;2-I

Technical Appendix Table. List of 12 immediately notifiable diseases included in the study of a mobile short-message-service–based disease outbreak alert system in Kenya*

Name of disease or event
Adverse events following immunization
Anthrax
Cholera
Dengue fever
Guinea worm
Measles
Neonatal tetanus
Plague
Rift Valley fever
Viral hemorrhagic fever
Yellow fever
Any public health event of international concern (e.g., infectious, zoonotic, foodborne, chemical, radionuclear, or caused by an unknown condition)

*The 12 diseases and conditions were selected from the World Health Organization's Integrated Disease Surveillance and Response national guidelines for Kenya.
